# Risk Factors for Unilateral Trigeminal Neuralgia Based on Machine Learning

**DOI:** 10.3389/fneur.2022.862973

**Published:** 2022-04-08

**Authors:** Xiuhong Ge, Luoyu Wang, Lei Pan, Haiqi Ye, Xiaofen Zhu, Qi Feng, Zhongxiang Ding

**Affiliations:** ^1^Department of Radiology, Hangzhou First People's Hospital, Zhejiang University School of Medicine, Hangzhou, China; ^2^Centre for Cognition and Brain Disorders, The Affiliated Hospital of Hangzhou Normal University, Hangzhou, China; ^3^Department of Radiology, Key Laboratory of Clinical Cancer Pharmacology and Toxicology Research of Zhejiang Province, Affiliated Hangzhou First People's Hospital, Cancer Center, Zhejiang University School of Medicine, Hangzhou, China

**Keywords:** machine learning, neurovascular compression, risk factors, classical trigeminal neuralgia, idiopathic trigeminal neuralgia

## Abstract

**Purpose:**

Neurovascular compression (NVC) is considered as the main factor leading to the classical trigeminal neuralgia (CTN), and a part of idiopathic TN (ITN) may be caused by NVC (ITN-nvc). This study aimed to explore the risk factors for unilateral CTN or ITN-nvc (UC-ITN), which have bilateral NVC, using machine learning (ML).

**Methods:**

A total of 89 patients with UC-ITN were recruited prospectively. According to whether there was NVC on the unaffected side, patients with UC-ITN were divided into two groups. All patients underwent a magnetic resonance imaging (MRI) scan. The bilateral cisternal segment of the trigeminal nerve was manually delineated, which avoided the offending vessel (Ofv), and the features were extracted. Dimensionality reduction, feature selection, model construction, and model evaluation were performed step-by-step.

**Results:**

Four textural features with greater weight were selected in patients with UC-ITN without NVC on the unaffected side. For UC-ITN patients with NVC on the unaffected side, six textural features with greater weight were selected. The textural features (rad_score) showed significant differences between the affected and unaffected sides (*p* < 0.05). The nomogram model had optimal diagnostic power, and the area under the curve (AUC) in the training and validation cohorts was 0.76 and 0.77, respectively. The Ofv and rad_score were the risk factors for UC-ITN according to nomogram.

**Conclusion:**

Besides NVC, the texture features of trigeminal-nerve cisternal segment and Ofv were also the risk factors for UC-ITN. These findings provided a basis for further exploration of the microscopic etiology of UC-ITN.

## Introduction

Trigeminal neuralgia (TN) is a chronic neurogenic pain (1, 2), also known as the most severe pain that humans can withstand. The pain can be triggered by harmless stimuli, such as chewing, brushing, and gently touching the face, or occur autonomously. It is characterized by intermittent paroxysmal facial shock or tingling, often involving one or more branches of the trigeminal nerve, with the second and third branches of the trigeminal nerve being more frequent (3). TN is mainly unilateral, and bilateral TN is rare (4).

Based on etiology, TN is mainly divided into classical TN (CTN), secondary TN (STN), and idiopathic TN (ITN). Of these, CTN is the most common type, accounting for about 85% (4). The pathogenesis of CTN is not clear. At present, it is generally accepted that “neurovascular compression (NVC)” is the mainstream cause (5). That is, the offending vascular (Ofv) compresses the trigeminal-nerve root entry zone, resulting in the demyelination and axonal loss of the trigeminal nerve, hence triggering a short circuit between pain fibers and non-pain fibers.

According to the International Classification of Headache Disorders 3rd edition (ICHD-3), when TN is not associated with the evidence of morphological changes in the nerve root and CTN cannot be defined, the condition is considered ITN. In this study, we called them ITN-nvc (6). We speculated that NVC might be one pathogenicity of ITN-nvc.

Although it was generally accepted that NVC was the main cause of CTN, NVC was also present in healthy individuals (7), even on the unaffected side of CTN (8, 9). Hence, NVC could lead to CTN, but it was not considered the sole risk factor.

Neurovascular compression not only leads to macrostructural abnormalities in the cisternal segment of the trigeminal nerve, such as the distortion and displacement of trigeminal nerve (10) and the thinning of a nerve at the compression point, but also causes microstructural changes, such as decreased fractional anisotropy (FA) and increased axial diffusion index of the trigeminal nerve in CTN (11). This study aimed to investigate the differences in the microstructure of trigeminal-nerve cistern segment between the affected and unaffected side in patients with unilateral CTN or ITN-nvc (UC-ITN) by machine learning (ML), and to explore the risk factors for UC-ITN, thus providing a basis for further clarification of the causes of disease.

## Methods

### Study Design and Patients

This was a prospective cross-sectional study as a part of an ongoing observational longitudinal trial, aiming to explore the risk factors for UC-ITN by ML, following “EQUATOR Reporting Guidelines” (12). This study was approved by the ethics committee of Hangzhou First People's Hospital, Zhejiang University School of Medicine, China (IRB# NO.202107002).

### Sample Size Estimation

In our study, the null hypothesis (H_0_) is that the parameters of trigeminal nerve cisternal segment have no significant effect on the affected side (β_1_ = 0), and an alternative hypothesis (H_1_) is that the parameters of trigeminal nerve cisternal segment have significant effect on the affected side (β_1_ ≠ 0). Given the conditional probability *p*_1_: = *p*(Y = 1 | X = 1) under H_0_ and *p*_2_: = *p*(Y = 1 | X = 1) under H_1_, we define the effect size (odds ratio, *OR*) by *p*_1_ and *p*_2_, *OR*: = [*p*_2_/(1 – *p*_2_)]/[*p*_1_/(1 – *p*_1_)]. The parameters β_0_ and β_1_ are related to *p*_1_ and *p*_2_ as follows: β_0_ = ln[*p*_1_/(1 – *p*_1_)], β_1_ = ln[*OR*] (Note: β_1_, is the regression coefficient of the independent variable. β_0_, is the constant term of logistic regression. *OR* was estimated by *p*_1_ = 0.5 and *p*_2_ = 0.3, which were recommended in GPower). According to the binary logistic regression analysis, we use the GPower software to estimate the sample size. The power level (1 – β) = 0.80, α = 0.05, and *OR* = 2.33 (which was calculated in the second step), the results show that at least 67 patients need to be included. The power level (1 – β) = 0.90, α = 0.05, and *OR* = 2.33, the results show that at least 86 patients need to be included. In our study, the sample size is 89, which is greater than the sample size when the 1 – β = 0.90. So, the sample size is adequate.

Patients with TN attending Hangzhou First People's Hospital, Zhejiang University School of Medicine from July 2021 to December 2021 were enrolled.

The inclusion criteria were as follows: (1) patients diagnosed with CTN or ITN-nvc, according to ICHD-3 (6, 13) criteria; (2) patients with unilateral CTN or ITN-nvc; and (3) patients underwent MRI scan with complete sequence, such as 3D volume interpolation body part examination (3D-VIBE) and 3D short time inversion recovery (3D-STIR).

The exclusion criteria were as follows: (1) patients with UC-ITN who received surgical treatment; (2) patients with STN or ITN but without NVC; and (3) poor image quality affecting the analysis.

Finally, 89 patients with UC-ITN were included (Table 1) and divided into two groups: patient group with NVC on the unaffected side of UC-ITN, and patient group without NVC on the unaffected side of UC-ITN.

**Table 1:** Demographic and clinical characteristics of patients with CTN.

		**UC-ITN without NVC on the unaffected side**	**UC-ITN with NVC on the unaffected side**	
			**Affected side**	**Unaffected side**
Number		38.20% (34/89)	61.80% (55/89)	
Age (year)		62 (52–68)	59 (53–69)	
Sex	Male	38.24% (13/34)	38.19% (21/55)	
	Female	61.76% (21/34)	61.82% (34/55)	
Side of CTN	R	64.71% (22/34)	67.27% (37/55)	
	L	35.29% (12/34)	32.73% (18/55)	
Distribution of CTN	V2.3	38.24% (13/34)	34.55% (19/55)	
	V3	20.59% (7/34)	30.91% (17/55)	
	V2	17.65% (6/34)	20.00% (11/55)	
	V1.2.3	8.82% (3/34)	5.45% (3/55)	
	V1.2	5.88% (2/34)	9.09% (5/55)	
	V1	5.88% (2/34)	NA	
	V1.3	2.94% (1/34)	NA	
BNI score	2	8.82% (3/34)	1.82% (1/55)	
	3	47.06% (16/34)	29.09% (16/55)	
	4	38.24% (13/34)	60.00% (33/55)	
	5	5.88% (2/34)	9.09% (5/55)	
Ofv	SCA	55.88% (19/34)	75.55% (41/55)	89.09% (49 /55)
	ASCA	14.71% (5/34)	7.27% (4/55)	7.27% (4/55)
	Others	29.41% (10/34)	18.18% (10/55)	3.64% (2/55)
POfvC	Proximal	38.24% (13/34)	30.91% (17/55)	29.09% (16/55)
	Distal	61.76% (21/34)	69.09% (38/55)	70.91% (39/55)
DOfvC	Class 1	44.12% (15/34)	36.36% (20/55)	45.45% (25/55)
	Class 2	47.06% (16/34)	52.73% (29/55)	54.55% (30/55)
	Class 3	8.82% (3/34)	10.91% (6/55)	/

*CTN, Classical trigeminal neuralgia; NVC, neurovascular compression; UC-ITN, unilateral classical trigeminal neuralgia or idiopathic trigeminal neuralgia with NVC; SCA, superior cerebellar artery; ASCA, anterior superior cerebellar artery; AICA, anterior inferior cerebellar artery; VA, vertebral artery; BA, basilar artery; TAHI, tiny arteries that are hard to identify; Ofv, offending vessel; POfvC, the position of offending vascular compression; DOfvC, the degree of offending vascular compression*.

### Imaging Protocol

All patients underwent MRI using a 3.0 T MRI scanner (Siemens, MAGNETOM Verio, Germany) and an eight-channel phased-array head coil. Trigeminal 3D-VIBE data were acquired using the following parameters: TR = 10 ms; TE = 3.69 ms; flip angle = 12 degrees; field of view (FOV) = 220 mm × 220 mm; voxel size = 0.8 mm × 0.8 mm × 0.8 mm; slice thickness = 0.8 mm; 60 slices) and 3D-STIR data acquisition (SPC sequence; TR = 3,800 ms; TE = 194 ms; FOV = 230 mm × 230 mm; voxel size = 0.9 mm × 0.9 mm × 0.9 mm; slice thickness = 0.9 mm; 64 slices).

### Trigeminal Structure

**Figure 1:**
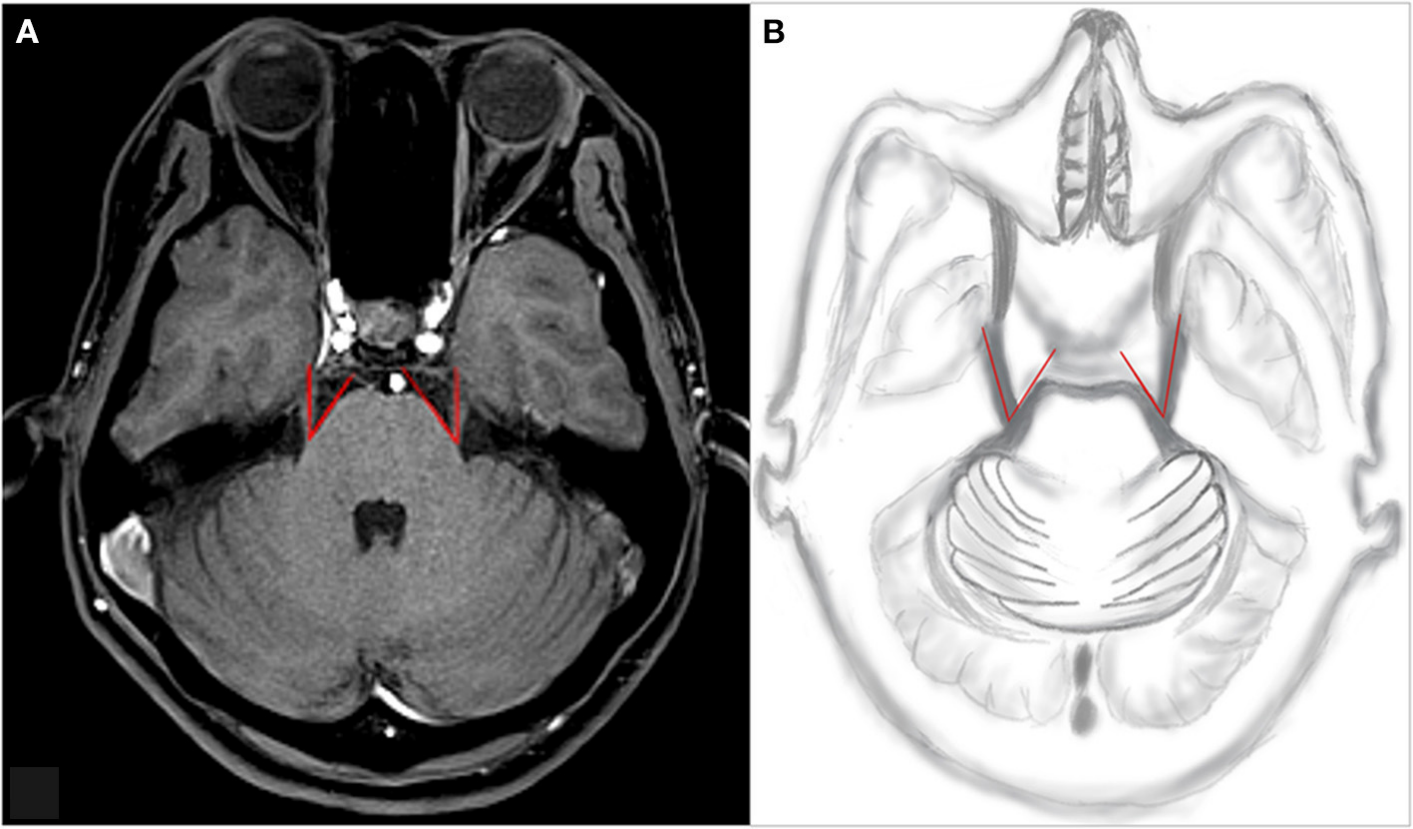
An example of TPA measurement. Along the main axis of the trigeminal nerve, the point emanating from the brainstem through the trigeminal nerve is tangential to the pons, and the angle is composed of the two lines. **(A)** An example of TPA measurement of the patient and **(B)** TPA drawn by hand. TPA, trigeminal pons angle.

Original images of 3D-VIBE and 3D-STIR sequences were transmitted to the Siemens postprocessing workstation for analysis. Trigeminal pons angle (TPA) was measured separately by a junior physician with 3 years of experience and a senior physician with 8 years of experience. Along the main axis of the trigeminal nerve, the point emanating from the brainstem through the trigeminal nerve was tangential to the pons, and the angle was composed of two lines (14) (Figure 1). Ofv, the position of Ofv compression (POfvC), and the degree of Ofv compression (DOfvC) were judged. “Posterior” of the POfvC meant that the Ofv was located in the region along the cisternal segment of the trigeminal nerve near one-third of the length of the pons. “Anterior” meant that the Ofv was located in the region along the cisternal segment of the trigeminal nerve distal two-thirds of the length of the distal pons. DOfvC was divided into four classes. Class 0, no relationship was found between the nerve and the vessel, or the relationship was blurred and difficult to evaluate. Class 1, the vessel crossed or touched the nerve without any visible layer of the cerebrospinal fluid or any deformity of the root. Class 2, the significant indentation of the root was found, which was caused by the compression of responsible vessel. Class 3, nerve distortion and/or displacement occurred (10).

### Radiomics Analysis

To clarify the differences between the affected and unaffected side of the trigeminal nerve, we performed the texture analysis on bilateral cisternal segment of trigeminal nerve.

**Figure 2:**
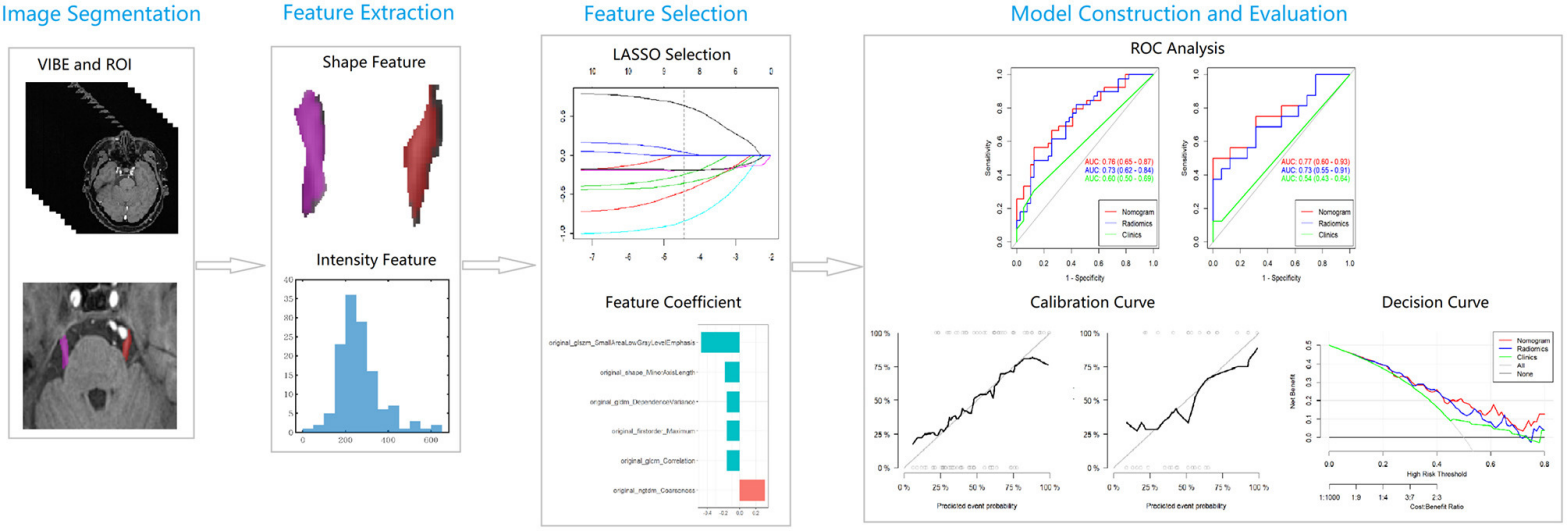
A flowchart of radiomics analysis. LASSO, least absolute shrinkage and selection operator; ROC, receiver operating characteristic curve.

The original image of the 3D-VIBE sequence and clinical parameters were uploaded to uAI Research Portal (United Imaging Intelligence, China) that was embedded into the widely used package PyRadiomics (https://pyradiomics.readthedocs.io/en/latest/index.html). The study population was assigned with the method of 10-fold cross-validation to avoid the sample bias of grouping. Statistical software R. (V.3.6.1, Vienna, Austria) was used for feature selection and nomogram generation. ML steps were as follows (Figure 2):

1. Delineation of the region of interest. The region of interest (ROI) was delineated, and the trigeminal cisternal segment was manually delineated layer by layer (from the location where the nerve exits the pons to a pre-determined boundary at the entrance of the Meckel's cave) on 3D-VIBE images, avoiding the Ofv.

2. Feature extraction. Based on the original images and ROI, the following features were extracted from the feature categories: the first-order, shape, Gray Level Co-occurrence Matrix (GLCM), Gray Level Run Length Matrix, Gray Level Size Zone Matrix, Gray Level Dependence Matrix, and Neighboring Gray Tone Difference Matrix, and the imaging filters.

3. Data grouping. The data were randomly divided into a training cohort (70%) and a validation cohort (30%).

4. Feature selection. Two feature selection methods, such as minimum redundancy maximum relevance (*mRMR)* and least absolute shrinkage and selection operator regression (*LASSO*) were used to select the features. First, *mRMR* was performed to eliminate the redundant and irrelevant features, and 10 features were retained. LASSO performance included choosing the regular parameter λ and determining the number of features. After the number of features was determined, the most predictive subset of features was chosen and the corresponding coefficients were evaluated.

5. Model construction. Rad_score was calculated by summing the selected features weighted by their coefficients and compared between the training and validation group. Subsequently, the radiomics score was incorporated with independent clinico-radiological predictors to build the comprehensive nomogram for logistic regression.

6. Model evaluation. The discrimination was first quantified with the area under the curve (AUC) of the receiver operating characteristic (ROC) curve. Afterward, a calibration curve was used to estimate the coincidence between the prediction model and the actual outcomes. Finally, the decision curve was used to visualize the clinical net benefit of prediction models.

### Statistical Analysis

Statistical analysis was performed using the software SPSS 26.0 and R. Clinical and MRI morphological features were assessed using a chi-square test (for nominal variables) and Wilcoxon's test (for continuous variables). The characteristics with a *p* < 0.1 were analyzed by the univariate logistic regression to determine the risk factors. In addition, independent risk factors and optimal rad_scores were analyzed by multi-factor roadbed regression to establish the prediction nomogram. At the same time, collinearity was evaluated based on variance inflation factor (VIF), and the features with a VIF > 10 were deleted. The analysis of delineation consistency between the junior and senior physician was performed using the intra-class correlation coefficient (ICC).

## Results

### Intra-Class Correlation Coefficient

According to the ICC analysis, the image features, TPA, Ofv, POfvC, and DOfvC were found to be in good agreement (ICC ≥ 0.75). In this study, the results delineated by the senior physician for further analysis were chosen.

### Clinical Features

This study included 34 patients with UC-ITN but without NVC on the unaffected side and 55 patients with UC-ITN and NVC on the unaffected side. The main Ofv was superior cerebellar artery (SCA) (Table 1).

### Results of ML in UC-ITN Patients Without NVC on the Unaffected Side

**Figure 3:**
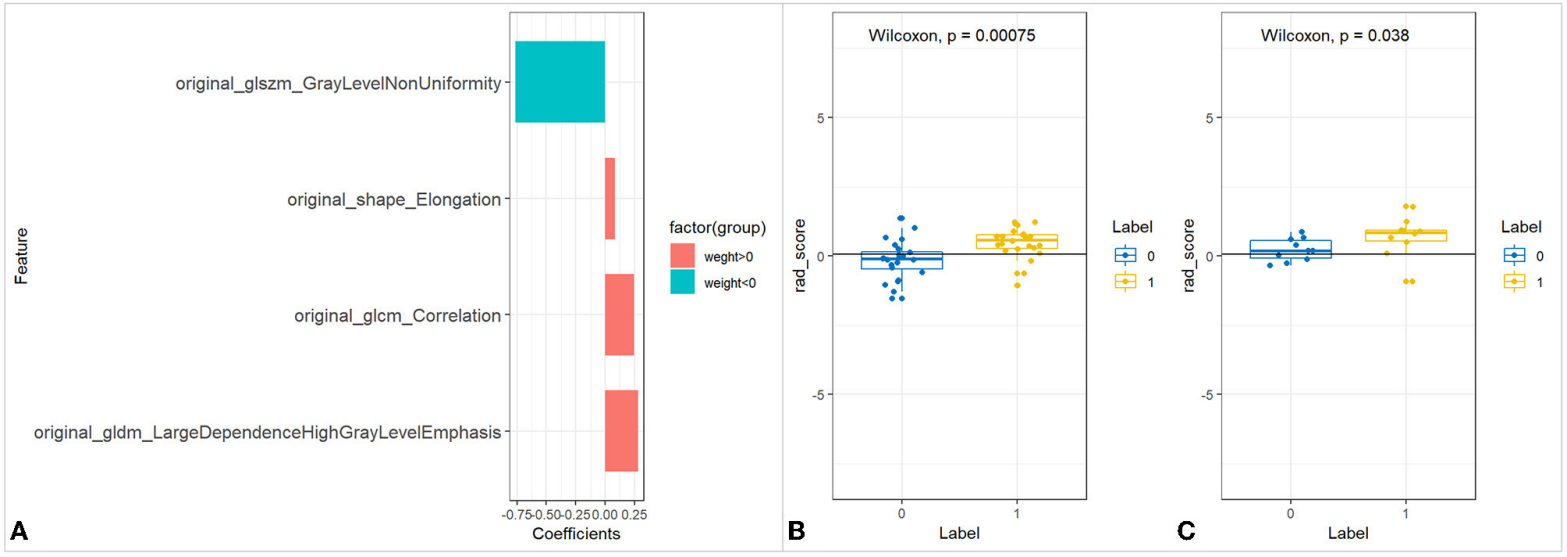
The feature selection of UC-ITN ROI without NVC on the unaffected side and its difference test. **(A)** Wilcoxon's test of the rad_score between the two groups of trigeminal-nerve cisternal segment feature selection and importance ratio. **(B,C)** Wilcoxon's test of rad_score in the training and validation cohorts. UC-ITN, unilateral classical Trigeminal neuralgia; ROI, region of interest; NVC, neurovascular compression.

A total of 117 features were selected from each ROI. Four textural features with greater weight, such as Gray Level Non-Uniformity (GLNU), Large Dependence High Gray Level Emphasis (LDHGLE), Correlation, and Elongation were selected. Rad_score showed statistically significant differences between the affected and unaffected sides (*p* < 0.05) (Figure 3).

### Results of ML in UC-ITN Patients With NVC on the Unaffected Side

#### Feature Selection

**Figure 4:**
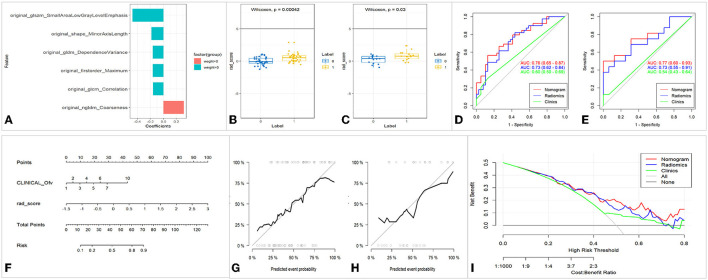
Feature selection, model construction, and evaluation of UC-ITN ROI with NVC on the unaffected side. **(A)** Wilcoxon's test of the rad_score between the two groups of trigeminal-nerve cisternal segment feature selection and importance ratio. **(B,C)** Wilcoxon's test of rad_score in the training and validation cohorts. **(D,E)** The ROC of the three models of the training and validation cohorts, respectively. **(F)** Nomogram. **(G,H)** Calibration curves of the training and validation cohorts, respectively. **(I)** Decision curve. ROC, receiver operating characteristic curve; 1, 2, 3, 4, 5, 6, 7, and 10 of the INCLINICAL_Ofv represent the superior cerebellar artery, anterior superior cerebellar artery, anterior inferior cerebellar artery, vertebral artery, basilar artery, superior cerebellar artery and anterior superior cerebellar artery, superior cerebellar artery and anterior inferior cerebellar artery, and tiny vascular that are hard to identify, respectively; Ofv, offending vascular; UC-ITN, unilateral classical Trigeminal neuralgia or idiopathic trigeminal neuralgia with NVC; ROI, region of interest; NVC, neurovascular compression.

A total of 117 features were selected from each ROI. Six textural features with greater weight, such as Small Area Low Gray Level Emphasis (SALGLE), Coarseness, Minor Axis Length (MAL) Dependence Variance (DV), Maximum, and Correlation were selected after dimensionality reduction using LASSO (Figure 4).

#### Model Construction

Rad_score was calculated by summing the selected features weighted by their coefficients. In the training and validation cohorts, rad_score was statistically significant (*p* < 0.05). Both the training and validation cohorts had consistent AUC, and the fit of the model was good (Figure 4). The nomogram model had better performance compared with the simple rad_score model and clinical characteristics model (Figure 4, Table 2). The nomogram was constructed, and the complex regression equation form was visually simplified (Figure 4).

**Table 2:** Parameters of the three models.

**Model**		**Accuracy**	**Sensitivity**	**Specificity**	**Pos. pred. value**	**Neg. pred. value**	**Pos. likeli**.	**Neg. likeli**.
		**(%)**	**(%)**	**(%)**	**(%)**	**(%)**	**ratios**	**ratios**
Radiomics	TC	69.23	82.05	56.41	65.31	75.86	1.88	0.32
	VC	68.75	68.75	68.75	68.75%	68.75	2.20	0.45
Clinics	TC	58.97	30.77	87.18	70.59	55.74	2.40	0.79
	VC	56.25	12.50	100.00	100.00	53.33	NA	0.88
Nomogram	TC	71.79	56.41	87.18	81.48	66.67	4.40	0.50
	VC	75.00	100.00	66.67	50.00	100.00	3.00	0.00

*TC, Training cohort; VT, Validation cohort*.

#### Model Evaluation

The calibration curves in the training and validation cohorts demonstrated a high diagnostic accuracy of the model (*p* = 0.40 vs. *p* = 0.79). Finally, the decision curve was used to evaluate the clinical usefulness of the model (Figure 4).

## Discussion

In our study, for UC-ITN patients without NVC on the unaffected side, only feature selection was performed and a model construction was not carried out. The reason was that there was no NVC on the affected side, which could be judged on the conventional image, and UC-ITN would not occur. The results showed that GLNU (15), LDHGLE (16), Correlation (17), and Elongation (18) had large weight in the cisternal segment of the trigeminal nerve. The reason may be that NVC led to trigeminal degeneration, demyelination, and morphological changes, causing the changes in the aforementioned features.

In this study, there are 55 UC-ITN patients with NVC on the unaffected side, indicating that the presence of NVC did not necessitate the UC-ITN condition. The results showed that SALGLE (19), Coarseness (20), MAL (21), DV (22), Maximum (23), Correlation (17), and the Ofv were the risk factors for UC-ITN with bilateral NVC. UC-ITN patients with bilateral NVC only suffered from unilateral pain, probably due to different degrees of trigeminal degeneration and demyelination between the affected and unaffected side, which might lead to changes in the above-mentioned texture features. The results demonstrated that the type of Ofv also played a role in the occurrence of UC-ITN with NVC on the unaffected side. Besides, Amaya Pascasio et al. (24) stated that the type of Ofv affected the prognosis of TN. Although the position of NVC on the root entry zoon was shown to be associated with TN development, anterior and posterior POfvC showed no statistical difference in our study. The reason might be that the sample size was small and all UC-ITN cases had NVC.

Although NVC was the mainstream theory of the etiology of CTN, the presence of NVC did not necessarily imply the occurrence of CTN. NVC was present in about 61.80% of the unaffected sides in patients with UC-ITN in this study, but no symptoms were presented. Arda et al. (8) demonstrated the presence of NVC on the unaffected side in patients with TN. Previous studies suggested that about 25–49% of healthy individuals (25, 26) and 14–39% of patients (27, 28) undergoing autopsy also had NVC that was consistent with our results.

In our study, 61.80% UC-ITN patients with NVC on the bilateral trigeminal nerve experienced only unilateral TN. What are the differences in the structure of the cisternal segment of the trigeminal nerve? Alper et al. (29) found that the cross-sectional area (CSA) of the cisternal segment of the trigeminal nerve was smaller in patients with TN compared with healthy controls (HC). It was speculated that CSA was a risk factor for TN, but the aforementioned study failed to clarify whether NVC occurred in HC. Moon et al. (11) found that compared with the healthy side, FA of the trigeminal nerve significantly decreased and the mean diffusion and RD in patients with TN. However, the number of patients included in the study was small. If the trigeminal nerve on the unaffected side with NVC was analyzed, it might provide a more reliable basis for the pathogenesis of CTN. Lee et al. (30) found that FA and quantitative anisotropy of the cisternal segment of the trigeminal nerve were lower on the unaffected side of patients with TN, and the trigeminal nerve volume on the unaffected side was smaller in patients with TN compared with HC. Wang et al. (5) found that the volume of the trigeminal nerve was reduced in patients with CTN compared with HC. Although no significant difference was found in trigeminal volume between affected and unaffected sides in UC-ITN patients with NVC on the unaffected side in this study, it was possible that NVC was present in the bilateral trigeminal nerve of patients with UC-ITN. These findings might provide a more reliable basis for studying the etiology of UC-ITN, suggesting a microscopic difference in the trigeminal nerve but no significant difference in the gross structure. Hardaway et al. (7) found that the mean anteroposterior diameter of the anterior pontine cistern and the length of the trigeminal nerve were smaller in patients with TN compared with HC. In addition, the posterior fossa volume was significantly smaller in TN patients without NVC compared with TN patients with NVC, suggesting that the posterior fossa volume played a role in the pathogenesis of TN. However, the study failed to classify patients and compare with HC by the presence or absence of NVC. Moreover, the above studies used different methods to characterize TN, not including ML.

The application of ML in the medical sector have witnessed a continuous increase in recent years, aiming to support the clinical decision-making (31). ML have been used in TN study to explore the morphological features of trigeminal nerve, pathogenesis, and prognostic factor and so on. In our study, the differences in texture features were found in both the affected and unaffected sides of the trigeminal nerve cisternal segments. Besides, Lin et al. (32) analyzed the flatness feature of Meckel's cave (MC) using the radiomics method. They found that the affected side of the primary TN (PTN) was lower than that of the unaffected side, as was the PTN without bilateral NVC, the right side of PTN and HC was lower than that of the left side, and the affected side was lower than that of the unaffected side. Mulford et al. (33) found that the radiomics intensity and texture features of the trigeminal nerves obtained from MR imaging were correlated with the presence of pain in TN. Danyluk et al. (1) used texture features to analyze T1-weighted MRI images in patients with CTN. They found that there were significant texture abnormalities in several pain-relevant brain regions that could segregate TN from HC.

This study had certain shortcomings and limitations. First, it was a single-center study with certain biases. Future studies should be conducted in multiple centers, and the results should be verified. Second, the sample size of this study was small and this was a cross-sectional study. In the future, we will expand the sample size, conduct longitudinal study, and divide patients into groups according to the postoperative efficacy, we hope to determine TN patient subpopulations who may have good responses to surgery by ML. Third, this study only analyzed and examined the structure of the trigeminal-nerve cisternal segment. Future studies should comprehensively analyze and examine the trigeminal nerve to identify the risk factors for primary TN. Furthermore, we did not compare TN patients with HC and we will conduct this research in the future.

## Conclusions

This study verified that NVC could lead to UC-ITN, but the presence of NVC alone did not necessitate the occurrence of UC-ITN. The texture features of the cisternal segment of the trigeminal nerve, such as SALGLE, Coarseness, MAL, DV, Maximum, and Correlation, and the type of Ofv might be risk factors for UC-ITN. These findings provided some basis to further clarify the etiology of UC-ITN.

## Data Availability Statement

The raw data supporting the conclusions of this article will be made available by the authors, without undue reservation.

## Ethics Statement

This study was approved by the Ethics Committee of Hangzhou First People's Hospital, Zhejiang University School of Medicine (IRB# No. 202107002). The patients/participants provided their written informed consent to participate in this study. Written informed consent was obtained from the individual(s) for the publication of any potentially identifiable images or data included in this article.

## Author Contributions

ZD: work concept or design, make important revisions to the paper, and approved final paper for publication. XG: work concept or design, draft paper, data collection, and approved final paper for publication. LW and QF: data processing and approved final paper for publication. LP, HY, and XZ: data collection and approved final paper for publication. All authors contributed to the article and approved the submitted version.

## Funding

This study was supported by National Natural Science Foundation of China (81871337), Zhejiang Provincial Public Welfare Research Project (2021RC108, 2020RC092), Medical and Health Technology Project of Hangzhou (A20200507), and Hangzhou Agriculture and Social Development Scientific Research Guidance Project (20211231Y022).

## Conflict of Interest

The authors declare that the research was conducted in the absence of any commercial or financial relationships that could be construed as a potential conflict of interest.

## Publisher's Note

All claims expressed in this article are solely those of the authors and do not necessarily represent those of their affiliated organizations, or those of the publisher, the editors and the reviewers. Any product that may be evaluated in this article, or claim that may be made by its manufacturer, is not guaranteed or endorsed by the publisher.
